# Improvement of Gas Barrier Properties for Biodegradable Poly(butylene adipate-co-terephthalate) Nanocomposites with MXene Nanosheets via Biaxial Stretching

**DOI:** 10.3390/polym14030480

**Published:** 2022-01-25

**Authors:** Xionggang Wang, Xia Li, Lingna Cui, Yuejun Liu, Shuhong Fan

**Affiliations:** Key Laboratory of Advanced Packaging Materials and Technology of Hunan Province, School of Packaging and Materials Engineering, Hunan University of Technology, Zhuzhou 412007, China; cheersun@126.com (X.W.); xiali_2019@163.com (X.L.); lncui@hut.edu.cn (L.C.); liuyuejun@hut.edu.cn (Y.L.)

**Keywords:** PBAT, MXene, nanocomposite, gas barrier properties, biaxial stretching

## Abstract

In order to ease the white pollution problem, biodegradable packaging materials are highly demanded. In this work, the biodegradable poly (butylene adipate-co-terephthalate)/MXene (PBAT/Ti_3_C_2_T_X_) composite casting films were fabricated by melt mixing. Then, the obtained PBAT/Ti_3_C_2_T_X_ composite casting films were biaxially stretched at different stretching ratios so as to reduce the water vapor permeability rate (WVPR) and oxygen transmission rate (OTR). It was expected that the combination of Ti_3_C_2_T_X_ nanosheets and biaxial stretching could improve the water vapor and oxygen barrier performance of PBAT films. The scanning electron microscope (SEM) observation showed that the Ti_3_C_2_T_X_ nanosheets had good compatibility with the PBAT matrix. The presence of Ti_3_C_2_T_X_ acted as a nucleating agent to promote the crystallinity when the content was lower than 2 wt%. The mechanical tests showed that the incorporation of 1.0 wt% Ti_3_C_2_T_X_ improved the tensile stress, elongation at break, and Young’s modulus of the PBAT/Ti_3_C_2_T_X_ nanocomposite simultaneously, as compared with those of pure PBAT. The mechanical dynamical tests showed that the presence of Ti_3_C_2_T_X_ significantly improved the storage modulus of the PBAT nanocomposite in a glassy state. Compared with pure PBAT, PBAT-1.0 with 1.0 wt% Ti_3_C_2_T_X_ exhibited the lowest OTR of 782 cc/m^2^·day and 10.2 g/m^2^·day. The enhancement in gas barrier properties can be attributed to the presence of Ti_3_C_2_T_X_ nanosheets, which can increase the effective diffusion path length for gases. With the biaxial stretching, the OTR and WVPR of PBAT-1.0 were further reduced to 732 cc/m^2^·day and 6.5 g/m^2^·day, respectively. The PBAT composite films with enhanced water vapor and water barrier performance exhibit a potential application in green packaging.

## 1. Introduction

Green packaging materials are highly demanded in the recent years because of the ever-increasing plastic pollution problem. The traditional packaging materials, such as polyethylene (PE) and poly(vinyl chloride) (PVC), have been gradually replaced by biodegradable polymers such as poly(lactic acid) (PLA), poly(butylene adipate-co-terephthalate) (PBAT), and poly(butylene succinate) (PBS) [1,2,3,4]. Compared with other biodegradable polyesters, PBAT has adjustable properties due to the copolymerization of 1,4-butanediol, adipic acid, and terephthalic acid [5]. In addition, it has good ductility, good thermal resistance, and high impact performance, which is similar to PE. However, it was reported that the oxygen transmission rate (OTR) of PBAT under ambient conditions was around 1050 cc/m^2^·day, whereas the water vapor permeability rate (WVPR) was 3.3 × 10^−11^ g·m/m^2^·s·Pa, which made it difficult to meet the requirements for packaging applications [6,7,8]. The poor oxygen and water vapor barrier performances limit the broad applications of PBAT in packaging. Therefore, it is necessary to improve the oxygen and water vapor barrier performance of PBAT so as to prolong the shelf life and maintain good quality of food.

It is widely accepted that the incorporation of nanofillers is a simple and effective method to reduce the OTR and WVPR of polymer films [9,10,11,12] because the presence of fillers can have barrier effects that increase the escape distance of oxygen and water molecules [13,14]. Li et al. reported that the well-aligned graphene nanosheets simultaneously reduced the oxygen permeability and enhanced the aging resistance of the PBAT composite film [15]. The oxygen-containing groups on graphene nanosheets enhanced the interactions between water molecules and altered the diffusion paths of water molecules. Mondal et al. found that the WVPR of PBAT could be reduced by 25% with the addition of 4 wt% organically modified montmorillonite (OMMT) [16]. This was because the impermeable OMMT in the PBAT matrix increased the tortuosity of the path for water molecules. Li et al. mixed graphene nanosheets with PBAT by the solution casting method [15]. The presence of graphene resulted in an 80% reduction in water permeation and a 99% reduction in oxygen transmission of PBAT nanocomposite films, which was ascribed to the fact that the graphene nanosheets enlarged the effective diffusion path length of water and oxygen across the films.

MXene is a novel family of (2D) transition metal carbides and/or nitrides [17,18,19,20,21]. The abundance of functional groups on the surface of MXene, such as oxygen (=O), hydroxyl (-OH), or fluorine (-F), endows it with good compatibility with many polar polymer matrices [22]. MXene has attracted considerable research interest for various applications, such as energy storage, sensors, electromagnetic shielding, and so on [23,24,25,26]. Ti_3_C_2_T_X_ nanosheets have high stiffness and strength, which can serve as effective, reinforced fillers to improve the mechanical properties of polymer/Ti_3_C_2_T_X_ nanocomposites. In addition, Wu et al. demonstrated that a small amount of Ti_3_C_2_T_X_ improved the complex viscosity and storage modulus of PVDF nanocomposites significantly [22]. The ultrahigh molecular weight polyethylene (UMWPE) composites containing 0.75 wt% Ti_3_C_2_T_X_ had the best creep performance [27]. With the addition of 1.9 vol% of MXene, the Ti_3_C_2_T_X_/polystyrene nanocomposites exhibited a 54% higher storage modulus than that of neat polystyrene [28]. Yu et al. demonstrated that the addition of MXene improved the thermal stability and fire safety of polystyrene [29]. However, to the best of our knowledge, the PBAT/Ti_3_C_2_T_X_ nanocomposites have not been reported on yet. It is expected that the impermeable Ti_3_C_2_T_X_ nanosheets in the PBAT matrix via biaxial stretching can not only improve the gas barrier performance, but also enhance the thermal stability and stiffness of the nanocomposites.

Biaxial stretching processing is the process of stretching hot polymeric films in the cross-machine direction. It is also reported that the biaxial stretching process endows the polymer matrix with an ordered structure and improved gas barrier properties [30,31,32]. This is because the biaxial stretching can help to reduce the free volume of the amorphous region of the polymers, resulting in an enhancement in gas barrier performance [33]. In addition, the biaxial stretching can help the 2D filler in the polymer matrices form an orientation structure, which can benefit for the enhancement of the gas barrier performance [34]. Li et al. reported that the orientated OMMT in the PBAT matrix prepared by biaxial stretching significantly reduced the WVPR and caused a 99% reduction in OTR with an enhancement in elongation at break [15]. Yoksan et al. demonstrated that the stretched PLA/PBAT/thermoplastic starch composite films had stacked-layer planar morphology, which contributed to the improvement in crystallinity, impact strength, water vapor, and oxygen barrier properties [35].

## 2. Materials and Methods

### 2.1. Materials

Poly(butylene adipate-co-terephthalate) (PBAT) pellets were supplied by BASF. The melt flow index (190 °C, 2.16 kg) was 5.0 g/10 min. The MXene (Ti_3_C_2_T_X_, 400 mesh, purity > 99%) nanosheets etched by HF were supplied by 11 Technology Co., Ltd. (Changchun, China). The specific surface area of the obtained Ti_3_C_2_T_X_ was approximately 31.5 m^2^/g.

### 2.2. Preparation of PBAT/Ti_3_C_2_T_X_ Nanocomposite Biaxial Stretching Films

**Scheme 1:**
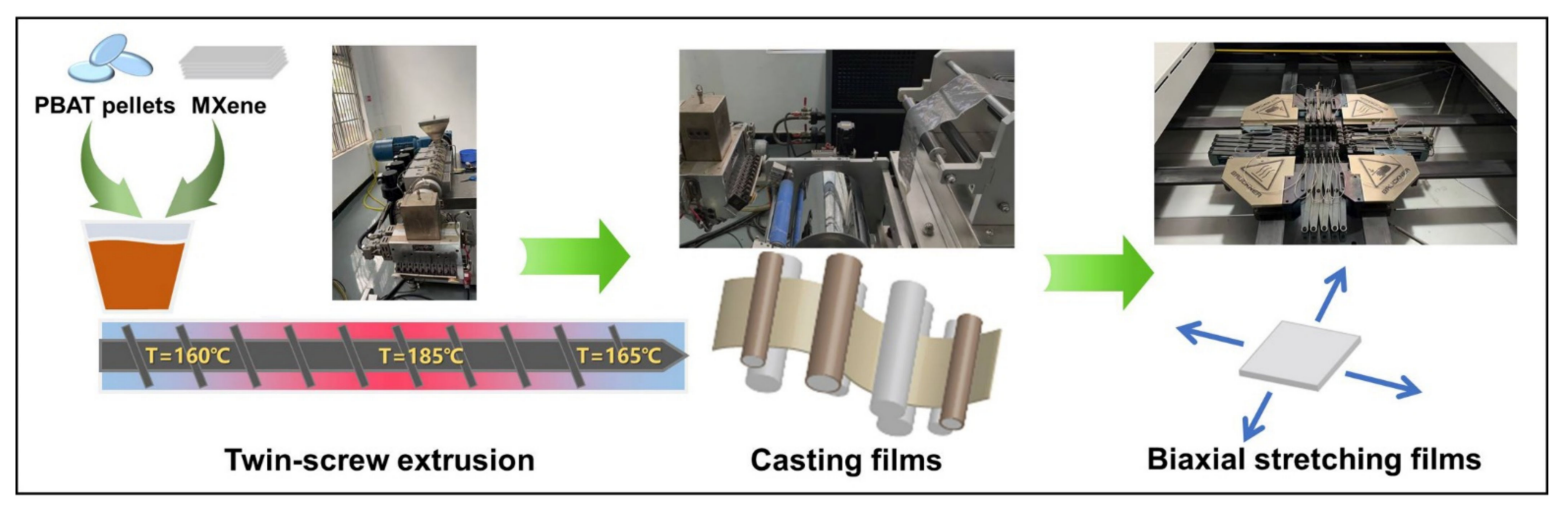
Schematic diagram of preparation of PBAT/Ti_3_C_2_T_X_ nanocomposite biaxial stretching films.

The preparation diagram of PBAT/Ti_3_C_2_T_X_ nanocomposite biaxial stretching films is shown in Scheme 1. To achieve a better dispersion of Ti_3_C_2_T_X_ nanosheets in the PBAT matrix, Ti_3_C_2_T_X_ was first mixed with PBAT pellets by melt compounding via a twin extruder to obtain composite casting films. Then the PBAT/Ti_3_C_2_T_X_ nanocomposite casting films were biaxially stretched. The effects of Ti_3_C_2_T_X_ content on the morphology, thermal stability, and crystallization behavior, as well as mechanical properties of PBAT/Ti_3_C_2_T_X_ nanocomposites, were comprehensively evaluated. The PBAT nanocomposite films containing the optimized 1 wt% Ti_3_C_2_T_X_ were biaxially stretched under different parameters.

The PBAT/Ti_3_C_2_T_X_ nanocomposite biaxial stretching films were prepared by two steps. Prior to the experiment, the PBAT pellets were dried in a vacuum oven at 80 °C for 12 h. Firstly, the PBAT/Ti_3_C_2_T_X_ nanocomposite casting film was prepared by extrusion casting (FDHU-35, Potpo, Guangzhou, China) so that the Ti_3_C_2_T_X_ could have a better dispersion in the PBAT matrix. The temperatures from the hopper to the nozzle were set at 130-150-170-170-170-150 °C. The screw speed was set at 60 rpm. The thickness of the obtained PBAT/Ti_3_C_2_T_X_ nanocomposite casting films was approximately 100 μm. The code of the samples was abbreviated as PBAT-X, where X represents the weight ratio of Ti_3_C_2_T_X_ in the PBAT/Ti_3_C_2_T_X_ nanocomposites.

The extruded PBAT nanocomposite casting films containing 1 wt% Ti_3_C_2_T_X_ nanosheets were cut into squares (80 mm side length) for biaxial stretching. The biaxially oriented films were then prepared at different stretching ratios on a KARO 5.0 (Brückner MaschinenbauGmbH & Co., Siegsdorf, Germany) equipped with mechanically driven clamps. The stretched films were thermally annealed at a temperature of 110 °C for 60 s. Finally, the biaxially oriented nanocomposite films were obtained to characterize the crystal orientation and the gas barrier properties.

### 2.3. Characterization

The fracture surfaces of the PBAT/Ti_3_C_2_T_X_ nanocomposite were characterized by scanning electron microscopy (SEM, Quanta 250, FEI, Hillsboro, OR, USA). The samples were fractured in liquid nitrogen for 30 min. They were observed at an accelerating voltage of 5 kV. Prior to the observation, all the samples were coated with a layer of gold.

Thermogravimetric analysis (TGA) of all PBAT/Ti_3_C_2_T_X_ nanocomposites was conducted on a TG-209F1 thermal analyzer (Netzsch, Selb, Germany) to measure the thermal stability under air atmosphere. The samples of about 10 mg were heated from room temperature to 600 °C at a heating rate of 10 °C/min.

The crystallization and melting behaviors of PBAT/Ti_3_C_2_T_X_ nanocomposites were conducted on a DSC-204F1 (Netzsch, Selb, Germany). The samples of approximately 5 mg were first heated to 180 °C at a heating rate of 10 °C/min to establish the thermal history, then cooled to 20 °C at a cooling rate of 10 °C/min and followed by a second heating rate of 10 °C/min to 180 °C. The peak crystallization temperature (*T*_cp_), the peak melting temperature (*T_m_*_p_), the crystallization enthalpy (Δ*H*_c_), and the melting enthalpy (Δ*H*_m_) of these samples were summarized. The degree of crystallinity of PBAT (*χ*_c_) was calculated by the following equation:

(1) $$\chi_{c}=\frac{\Delta H_{m}}{\Delta H_{0}\left(1-\varphi_{c}\right)}$$

where Δ*H*_0_ is the 100% melting enthalpy of PBAT (114 J/g) [36], and *φ*_c_ presents the weight ratio of Ti_3_C_2_T_X_ in the nanocomposites.

The tensile test was performed on an Instron 5566 universal electron tensile machine. The specimens were cut into rectangle shape with a dimension of 1 cm × 8 cm × 100 μm. The tensile speed was fixed at 10 mm/min. The reported results were the average values of at least five successful specimens.

The dynamical mechanical analysis was conducted on a Netzsch DMA 242E (Netzsch, Selb, Germany) analyzer. Tensile measurements were taken on specimens with dimensions of 30 mm at a fixed frequency of 1 Hz and from 90 °C to 70 °C at a ramping rate of 3 °C/min.

The 2D, wide-angle, X-ray scattering (2D-WAXS) measurements were carried out on an X-ray diffractometer (Rigaku UltimaIV, The Woodlands, TX, USA). The data were collected in the scanning range of 10–60° with a step of 0.02°.

The oxygen transmission rate (OTR) of the oriented films was measured with a MOCON OX-TRAN (Ametek Mocon, Brooklyn Park, MN, USA) 2/21 at 23 °C, 1 atm, and 85% RH. The water vapor transmission rate (WVTR) was determined according to ASTM E96-80 at MOCON PERMATRAN-W 3/33 (Ametek Mocon, Brooklyn Park, MN, USA). The reported values were the average results of three successful samples.

## 3. Results and Discussions

### 3.1. Morphology

**Figure 1:**
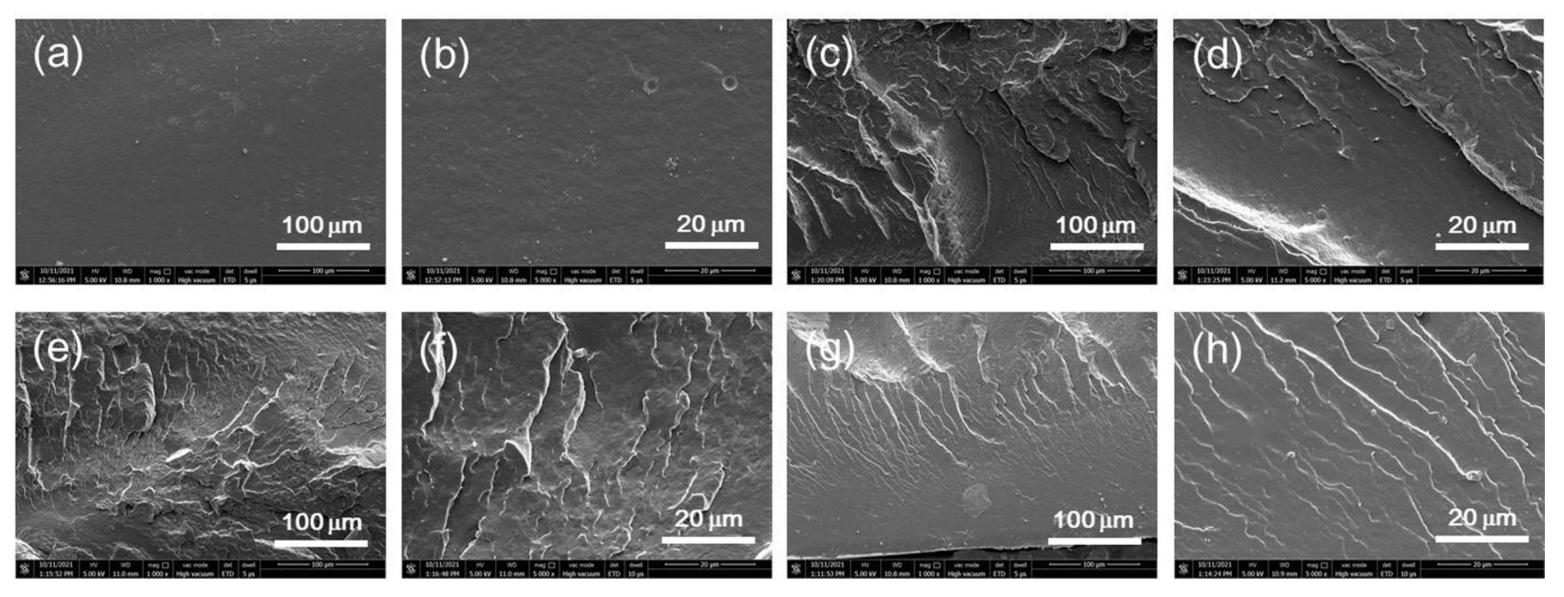
SEM images of the cross-section PBAT/Ti_3_C_2_T_X_ nanocomposite films. (**a**,**b**) PBAT-0, (**c**,**d**) PBAT-0.5, (**e**,**f**) PBAT-1.0, and (**g**,**h**) PBAT-2.0.

Figure 1 shows the SEM images of the cross-section fracture surfaces of PBAT/Ti_3_C_2_T_X_ nanocomposite films. In Figure 1a,b, PBAT-0 exhibits a relatively smooth surface due to its brittle fracture after immersion in liquid nitrogen [37]. With the addition of Ti_3_C_2_T_X_ nanosheets, the surfaces of the PBAT/Ti_3_C_2_T_X_ nanocomposites become gradually rough in Figure 1c–f when the Ti_3_C_2_T_X_ content is lower than 2.0 wt%. This is due to the presence of Ti_3_C_2_T_X_, which served as a rigid filler to transfer the stress during facture. In addition, it can be seen that an agglomeration phenomenon existed on the surface of PBAT-2.0 (Figure 1g,h). In Figure 1, no pores and holes are observable between the exposed Ti_3_C_2_T_X_ nanosheets on the surfaces (Figure 1f,h) and the PBAT matrix, indicating that Ti_3_C_2_T_X_ nanosheets have good compatibility with the PBAT matrix. It is attributed to the large number of polar groups of Ti_3_C_2_T_X_ that can react with the polyester groups of PBAT.

### 3.2. Thermal Stability

**Figure 2:**
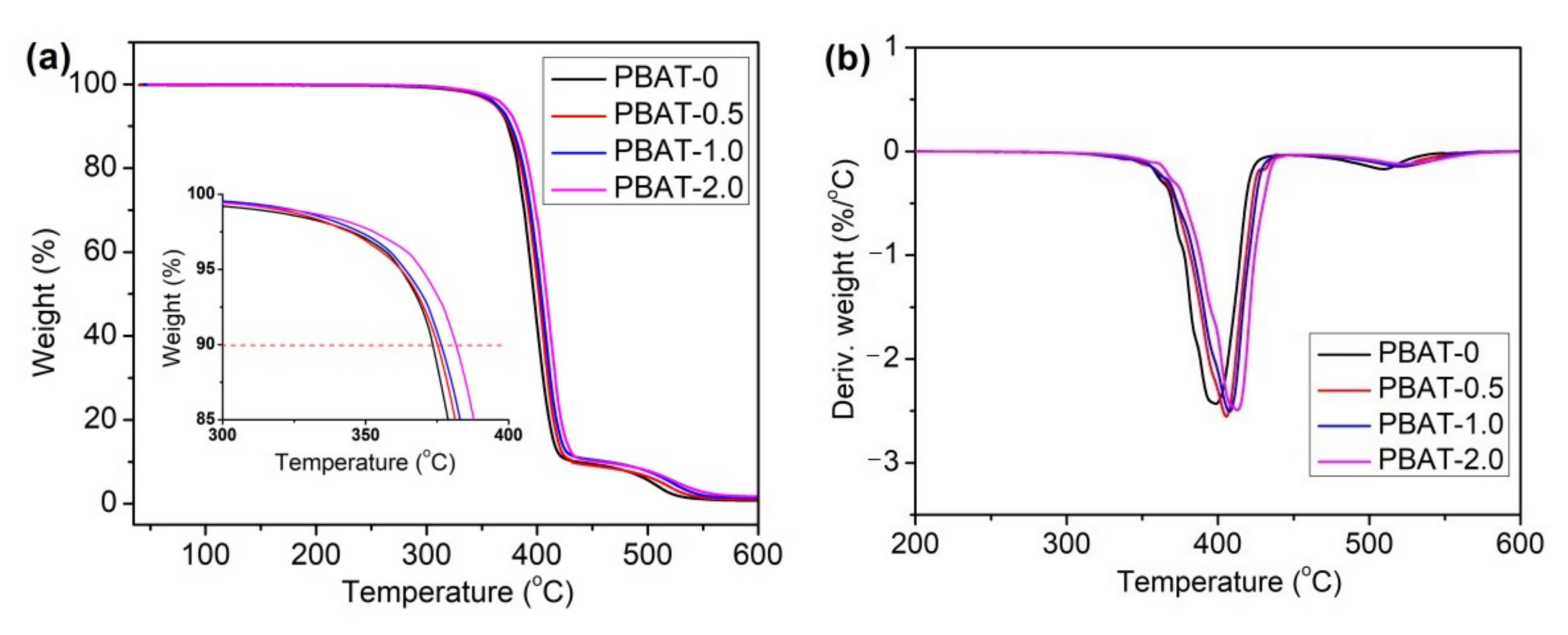
TGA curves of PBAT/Ti_3_C_2_T_X_ nanocomposites at air atmospheres. (**a**) TGA and (**b**) DTG.

The thermal stability of PBAT/Ti_3_C_2_T_X_ nanocomposites is shown in Figure 2, and the corresponding data, including the temperatures at 10% weight loss (*T*_10_), the temperatures at the maximum weight loss rate (*T*_max_), and the char yields at 600 °C, are listed in Table 1. In Figure 2a, two thermal decomposition stages are observable for all the samples. The first stage between 300 °C and 420 °C can be attributed to the random, main-chain scission and thermo-oxidative reactions of PBAT [38]. The second stage, in the range of 420–550 °C, corresponds to cis-elimination and thermo-oxidative reactions [32]. In Table 1, it can be seen that the values of *T*_10_ showed an increasing trend with the increase of Ti_3_C_2_T_X_ content. When compared to PBAT-0, the *T*_10_ of PBAT-2.0 dropped from 373.5 °C to 379.2 °C. In addition, the *T*_p_ of PBAT-2.0 gradually increased to 412.5 °C with the addition of 2 wt% Ti_3_C_2_T_X_. That is because the presence of Ti_3_C_2_T_X_ rapidly catalyzed the formation of a char layer that served as a thermal barrier to protect the underlying polymer matrices [39]. The improvement of char yield benefited from the isolating of volatile gases and oxygen; therefore, improving the thermal stability of PBAT. Furthermore, the char yield at 600 °C for PBAT-0, PBAT-0.5, PBAT-1.0, and PBAT-2.0 was 0.7%, 1.0%, 1.4%, and 1.7%, respectively. It was mainly due to the introduction of Ti_3_C_2_T_X,_ which promoted charring, and partial polymers could not be completely thermally decomposed, resulting in enhanced char residues [40].

### 3.3. Crystallization and Melting Behavior

**Figure 3:**
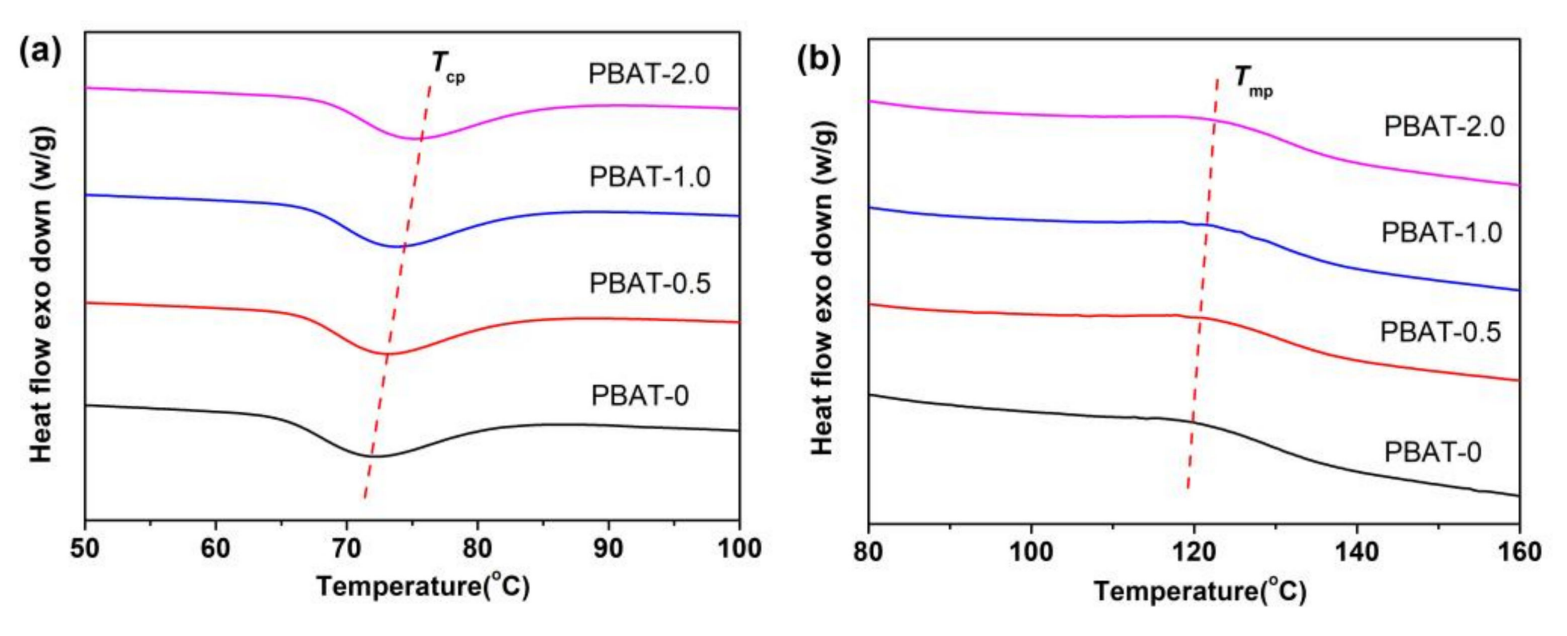
Differential scanning calorimetry (DSC) thermograms of PBAT/Ti_3_C_2_T_X_ nanocomposites. (**a**) first cooling, (**b**) second heating.

The DSC curves of the PBAT/Ti_3_C_2_T_X_ nanocomposites are shown in Figure 3. In Figure 3a, the onset crystallization temperatures of PBAT nanocomposites exhibit an increasing trend with the increase of Ti_3_C_2_T_X_ content. In addition, the values of *T*_cp_ for PBAT nanocomposites in Table 2 are 72.1, 73.1, 73.7, and 75.2 °C, respectively. The increase in *T*_cp_ indicated that the presence of Ti_3_C_2_T_X_ had a heterogeneous nucleation effect, accelerating the formation of crystallites in the PBAT matrix during cooling [41]. It was noted that the values of **Δ***H*_m_ were lower than **Δ***H*_c_ for the PBAT/Ti_3_C_2_T_X_ composites, which can be ascribed to the fast cooling rate. In Figure 3b, the values of *T*_mp_ for PBAT nanocomposites are 119.3, 121.0, 121.6, and 121.0 °C, respectively. The increase in *T*_mp_ suggests that the filling Ti_3_C_2_T_X_ contributes to the formation of perfect crystallinity of PBAT during the cooling procedure. Moreover, the crystallization degree of PBAT-1.0 had the highest value of 13.4% with the addition of 1.0 wt% Ti_3_C_2_T_X_. When the content of Ti_3_C_2_T_X_ was further increased up to 2.0 wt%, the crystallization degree of PBAT-2.0 showed a slight decrease. This may be due to the excessive addition of Ti_3_C_2_T_X_, which led to agglomeration, to some extent. On the other hand, the inhibition effects of the excessive Ti_3_C_2_T_X_ nanosheets were more profound than the nucleating effect that led to smaller crystallization and decreased crystallinity [42].

### 3.4. Mechanical Properties of Casting Films

**Figure 4:**
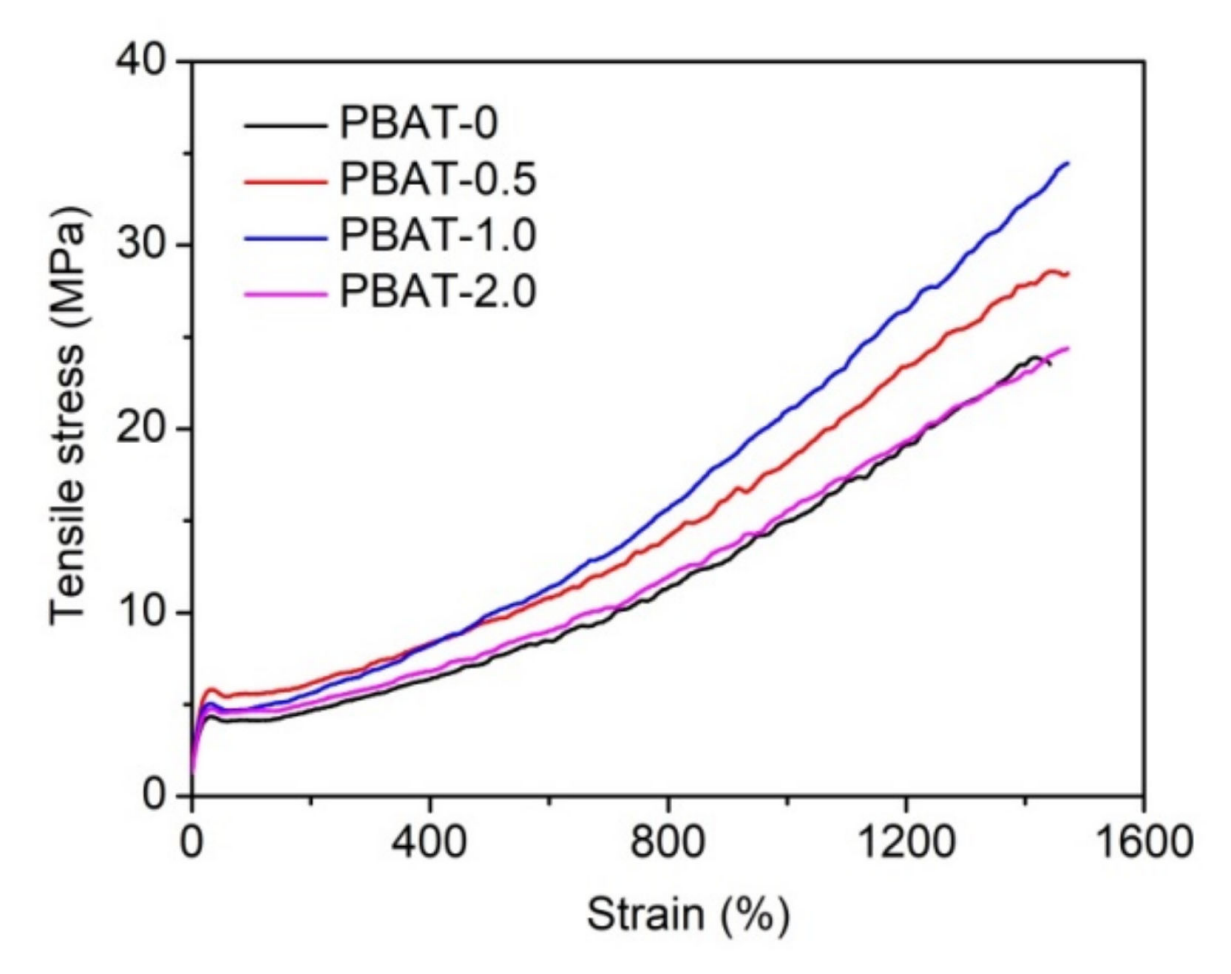
Tensile stress versus strain of PBAT/Ti_3_C_2_T_X_ nanocomposite casting films.

Figure 4 shows the typical stress–strain curves for pure PBAT and PBAT/Ti_3_C_2_T_X_ nanocomposite casting films, and the corresponding data are summarized in Table 3. It was observed that PBAT-0 exhibited a high ductility (elongation at break ~ 1442%) but low tensile strength (~22.6 MPa), which is consistent with previous report [14]. With the addition of 0.5 wt% Ti_3_C_2_T_X_, the tensile strength of the PBAT/Ti_3_C_2_T_X_ nanocomposite increased by 19.8% with a slight increase in the elongation at break. As depicted in Figure 1, the Ti_3_C_2_T_X_ had good interfacial interaction with the PBAT matrix; therefore, the addition of Ti_3_C_2_T_X_ nanosheets can transform the stress during the tensile process. When the Ti_3_C_2_T_X_ content was increased to 1.0%, the PBAT/Ti_3_C_2_T_X_ nanocomposite had the maximum tensile strength (31.6 MPa). This enhancement can be ascribed to the reinforcement effects of the nanofillers and the interaction between the stress concentration zones around the Ti_3_C_2_T_X_ nanosheets [43,44]. It is worth noting that PBAT-2.0 showed a decreasing tendency in both tensile stress and elongation at break as compared with PBAT-1.0. This may be due to the aggregation of Ti_3_C_2_T_X_ nanosheets in the PBAT matrix.

### 3.5. Dynamic Mechanical Analysis

**Figure 5:**
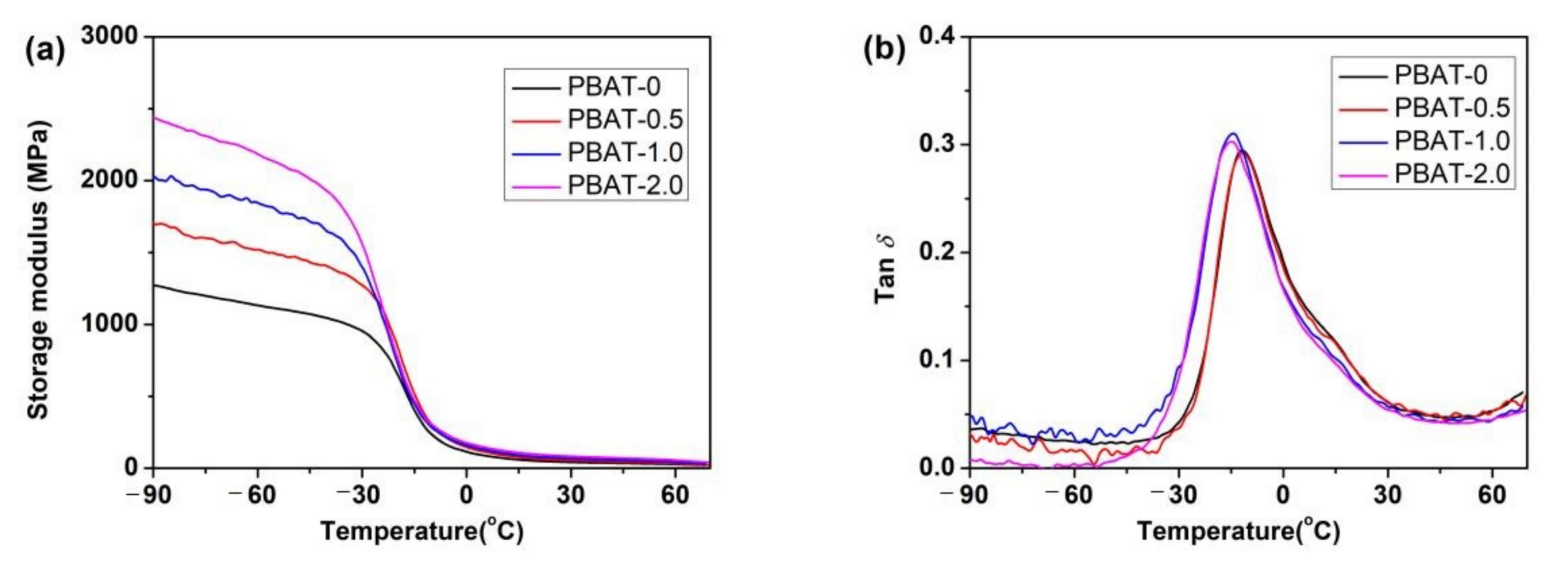
(**a**) Storage modulus and (**b**) loss factor of PBAT/Ti_3_C_2_T_X_ nanocomposite casting films.

The storage modulus as a function of temperature for PBAT/Ti_3_C_2_T_X_ nanocomposite casting films is shown in Figure 5a. It is clear that the storage modulus of the PBAT nanocomposite reinforced with Ti_3_C_2_T_X_ was higher than that of PBAT-0 in the glassy states. In addition, the reinforcement effect was more obvious with the increase of Ti_3_C_2_T_X_ content. When compared to PBAT-0, the storage modulus of PBAT-2.0 at 80 °C increased from 1220 MPa to 2342 MPa. This is due to the stiffening effect of rigid Ti_3_C_2_T_X_ nanosheets. Aside from this, the polar groups on the surface of Ti_3_C_2_T_X_ may have had intramolecular interaction with the PBAT. matrix, which may have also improved the storage modulus of the PBAT/Ti_3_C_2_T_X_ nanocomposites [43]. The loss factor peak (tan*δ*) is usually defined as the glass transition temperature (*T*_g_). It is observable from Figure 5b that the *T*_g_ shifted to a lower temperature when the PBAT matrix was incorporated with Ti_3_C_2_T_X_. With the addition of 2 wt% Ti_3_C_2_T_X_, the PBAT-2.0 shifted from −11.9 °C to −15.0 °C, as compared with that of PBAT-0. It can be attributed to the incorporation of Ti_3_C_2_T_X_, which can improve the chain mobility of the amorphous regions of PBAT due to the liberation effect of Ti_3_C_2_T_X_. In addition, the height of tan*δ* also showed a slight increase, indicating that an increase in Ti_3_C_2_T_X_ content will result in higher dissipative energy [45].

### 3.6. 2D-WAXS Patterns of Biaxial Stretching Films

**Figure 6:**
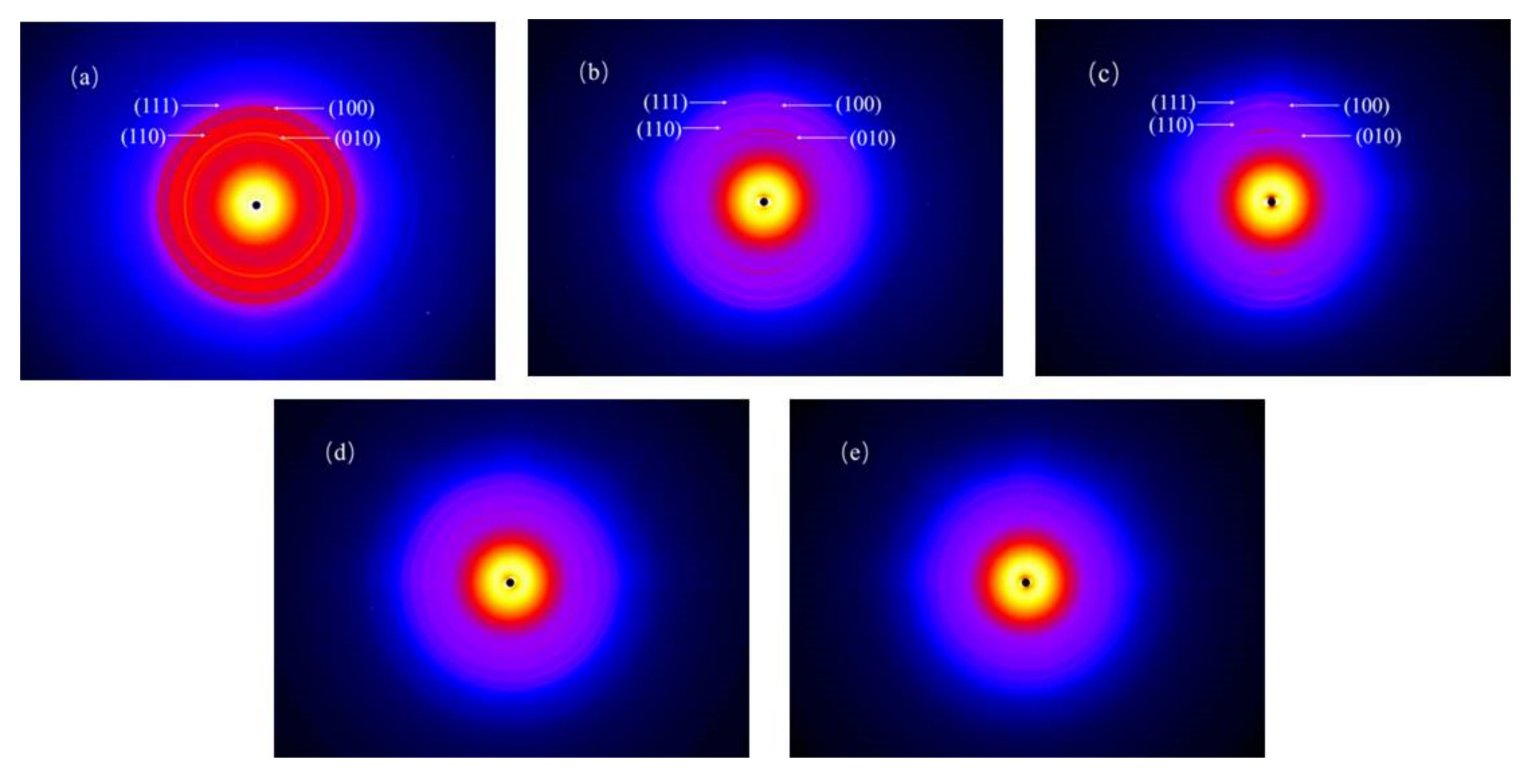
The films of PBAT-1.0 under different biaxial stretching ratio (transverse direction × machine direction). (**a**) 1 × 1, (**b**) 1 × 2, (**c**) 1 × 3, (**d**) 1.5 × 1.5, and (**e**) 2 × 2.

Figure 6 shows the 2D-WAXS images of the PBAT-1.0 casting films under different biaxial stretching ratios. It is observable that there are four crystal planes (111), (100), (110), and (010) in the PBAT film (1 × 1) in Figure 6a, and these crystal planes belong to the PBAT phase [10]. With the increase of the stretching ratio in the machine direction (MD), the crystal planes (111), (100), (110), and (010) in the PBAT composite films (Figure 6b,c) had more obvious orientation. In addition, the larger the stretching ratio, the more obvious the orientation effect, which indicates that uniaxial stretching can promote the orientation of the PBAT/Ti_3_C_2_T_X_ biaxial stretching films’ crystal form along the MD. In Figure 6d,e, there is no obvious crystal orientation in the 2D-WAXS diffraction pattern in the biaxially stretched PBAT/Ti_3_C_2_T_X_ films, indicating that the biaxial stretching will not cause the film to have an obvious crystal orientation in a certain direction. The crystal orientation of PBAT/Ti_3_C_2_T_X_ composite films further confirms that the biaxially oriented PBAT/Ti_3_C_2_T_X_ film has excellent isotropy.

### 3.7. Gas Barrier Properties of Biaxial Stretching Films

**Figure 7:**
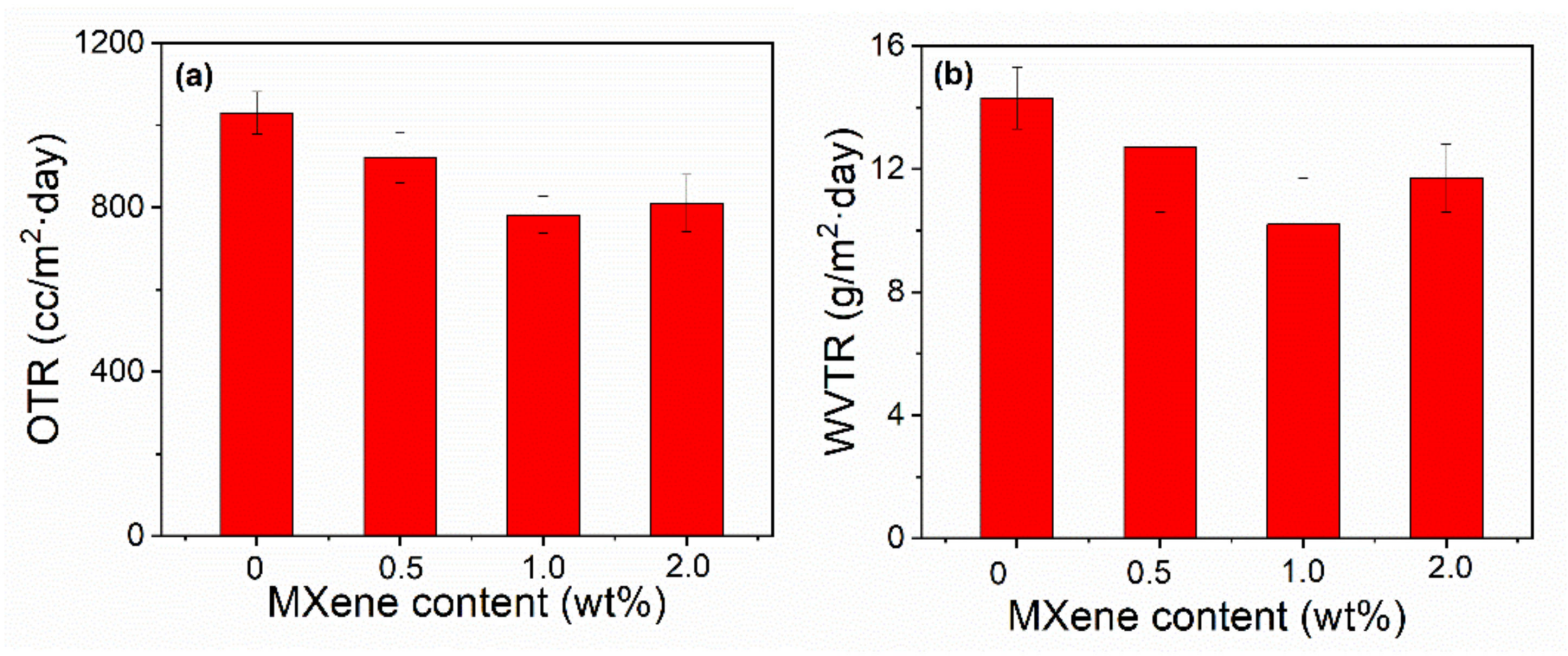
The gas barrier properties of PBAT/Ti_3_C_2_T_X_ nanocomposite casting films. (**a**) OTR, (**b**) WVTR.

The gas barrier properties of PBAT/Ti_3_C_2_T_X_ nanocomposite casting films are shown in Figure 7. In Figure 7a, the OTR of PBAT nanocomposite casting films shows a decreasing trend with the increase of Ti_3_C_2_T_X_ content. The lowest OTR was achieved for PBAT-1.0, which decreased from 1030 to 782 cc/m^2^·day. Similarly, the water vapor transmission rate (WVTR) of PBAT/Ti_3_C_2_T_X_ nanocomposite casting films decreased as the Ti_3_C_2_T_X_ content increased in the PBAT matrix. In Figure 7b, the WVTR for PBAT-0, PBAT-0.5, PBAT-1.0, and PBAT-2.0 is determined to be 14.3, 12.7, 10.2, and 11.7 g/m^2^·day, respectively. It is speculated that the addition of Ti_3_C_2_T_X_ nanosheets can serve as a barrier to form a tortuous path, increasing the effective diffusion path length. Furthermore, the abundant hydroxyl groups on the surface of Ti_3_C_2_T_X_ will contribute to the interactions with water molecules, delaying the diffusion to some extent. However, the aggregation of Ti_3_C_2_T_X_ will result in a deterioration of the gas barrier performance when the content of Ti_3_C_2_T_X_ is increased by up to 2.0 wt%.

**Figure 8:**
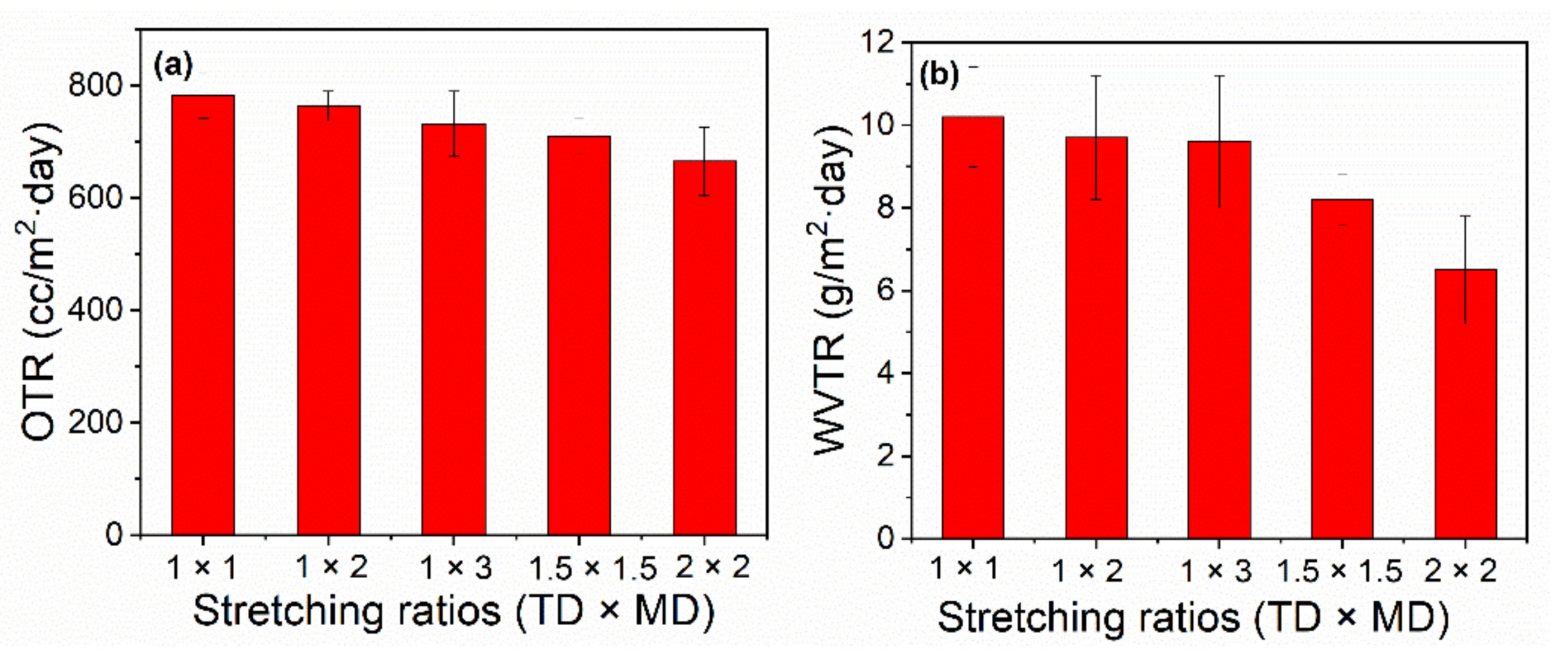
The gas barrier properties of PBAT-1.0 films under different biaxial stretching ratio. (**a**) OTR, (**b**) WVTR.

To investigate the effects of the stretching ratio on the gas barrier performance of PBAT/Ti_3_C_2_T_X_ nanocomposite stretching films, the OTR and WVTR data of PBAT-1.0 stretching film under different stretching ratios are shown in Figure 8. In Figure 8a, it is clear that the OTR of PBAT-1.0 stretching film decreased from 782 to 732 cc/m^2^·day with the stretching ratio increasing to 3 under uniaxial stretching. This can be attributed to the enhanced orientation of PBAT crystallites formed during the uniaxial stretching process, which is demonstrated in 2D-WAXS patterns (Figure 6). Meanwhile, the WVTR for PBAT-1.0 stretching film achieved the lowest value of 6.5 g/m^2^·day under 2 × 2 biaxial stretching condition, shown in Figure 8b. This is because the biaxial stretching process can contribute to the formation of an amorphous phase of PBAT and the exfoliation of Ti_3_C_2_T_X_ sheets. However, the barrier effect of Ti_3_C_2_T_X_ sheets is more profound than the effect of the PBAT amorphous phase, resulting in a further decrease in OTR and WVTR. The combination of two-dimensional, inorganic nanofillers with the biaxial stretching process paves the way for the preparation of a biodegradable polymer with enhanced gas barrier performance.

## 4. Conclusions

In this work, two-dimensional MXene (Ti_3_C_2_T_X_) nanosheets were mixed with PBAT by melt compounding. The effects of Ti_3_C_2_T_X_ content on the morphology, thermal stability, crystallization behavior, and gas barrier performance of PBAT were investigated. Furthermore, the effects of the biaxial stretching ratio on the gas barrier properties were further discussed. The TGA results showed that the addition of Ti_3_C_2_T_X_ improved the thermal stability of the PBAT nanocomposite. In addition, the tensile tests showed that the addition of 1.0 wt% Ti_3_C_2_T_X_ improved the maximum tensile stress without losing ductility. The storage modulus of PBAT was significantly improved in the glassy state with the addition of Ti_3_C_2_T_X_. After biaxial stretching, the PBAT-1.0 film (1 × 3) exhibited an oxygen transmission rate of 732 cc/m^2^·day, which was 28.9% lower than that of pure PBAT casting film. When the stretching ratio was 2 × 2, the WVTR of PBAT-1.0 biaxial stretching film was 6.5 g/m^2^·day, which was 36.3% lower than that of 1 × 1 PBAT-1.0 film. The enhancement in gas barrier properties can be attributed to the presence of Ti_3_C_2_T_X_ nanosheets, which can increase the effective diffusion path length for gases. The results of this work indicate the need for further studies on the influence of the orientation and surface functionalization of Ti_3_C_2_T_X_ nanosheets, as well as the incorporation of compatbilizers in the PBAT composite films for packaging applications.

## Figures and Tables

**Table 1:** Thermal stability of PBAT/Ti_3_C_2_T_X_ nanocomposite films.

Samples	*T*_10_ (°C)	*T*_p_ (°C)	Char Yield at 600 °C (wt%)
PBAT-0	373.5	398.3	0.7
PBAT-0.5	375.1	404.7	1.0
PBAT-1.0	376.4	408.5	1.4
PBAT-2.0	379.2	412.5	1.7

**Table 2:** DSC thermograms of PBAT/Ti_3_C_2_T_X_ nanocomposites.

Samples	*T*_cp_ (°C)	Δ*H*_c_ (J/g)	*T_m_*_p_ (°C)	Δ*H*_m_ (J/g)	*χ* (%)
PBAT-0	72.1	16.8	119.3	14.6	12.8
PBAT-0.5	73.1	17.0	121.0	14.7	13.0
PBAT-1.0	73.7	16.8	121.6	15.1	13.4
PBAT-2.0	75.2	16.9	121.0	14.2	12.7

**Table 3:** Tensile properties of PBAT/Ti_3_C_2_T_X_ nanocomposite casting films.

Samples	Tensile Stress (MPa)	Young’s Modulus (MPa)	Elongation at Break (%)
PBAT-0	22.6 ± 3.2	24.5 ± 4.1	1442.3 ± 104.5
PBAT-0.5	27.1 ± 3.6	28.6 ± 2.0	1524.8 ± 98.7
PBAT-1.0	31.6 ± 4.7	31.4 ± 2.9	1483.2 ± 132.4
PBAT-2.0	24.3 ± 5.1	32.1 ± 3.3	1350.3 ± 329.7

## Data Availability

The raw/processed data required to reproduce these findings cannot be shared at this time as the data also form part of an ongoing study.
